# Detailed Insights into the Relationship Between Three-Dimensional Speckle-Tracking Echocardiography-Derived Systolic Left Atrial Global Strains and Left Ventricular Volumes in Healthy Adults from the MAGYAR-Healthy Study

**DOI:** 10.3390/jcm14041143

**Published:** 2025-02-10

**Authors:** Attila Nemes, Nóra Ambrus, Csaba Lengyel

**Affiliations:** Department of Medicine, Albert Szent-Györgyi Medical School, University of Szeged, H-6725 Szeged, Hungary; ambrusnora@gmail.com (N.A.); lecs@in1st.szote.u-szeged.hu (C.L.)

**Keywords:** left atrial, left ventricular, volume, strain, speckle tracking, three dimensional, echocardiography

## Abstract

**Background and Objectives:** The complex relationship between three-dimensional (3D) speckle-tracking echocardiography (3DSTE)-derived left ventricular (LV) and left atrial (LA) volumes and functional properties has been demonstrated in recent studies. A better understanding of LV volumetric dependence on systolic peak LA (reservoir) strains in healthy circumstances could complete this knowledge. Therefore, 3DSTE was used for the simultaneous evaluation of these parameters in healthy adults, aiming to examine their complex relationship. **Materials and Methods:** The present study consisted of 165 healthy individuals with a mean age of 33.1 ± 12.3 years and 90 men. A complete two-dimensional echocardiography with Doppler with 3DSTE was performed in all the cases. **Results:** The peak LA global radial (GRS), longitudinal (GLS), and 3D (G3DS) strains were increased in the subjects with a mean LV end-diastolic volume (EDV) as compared to those cases with a lower-than-mean LV-EDV. In the cases with a higher-than-mean LV-EDV, no further increase in these peak global LA strains could be detected. The peak LA global circumferential and area strains showed a tendentious (non-significant) increase with an increasing LV-EDV. The peak LA global strains showed similar non-significant associations with the LV end-systolic volume (except the peak LA-G3DS, which proved to be significant). **Conclusions:** In healthy adults, the 3DSTE-derived peak LA-GRS and LA-G3DS are increased with a larger LV-EDV up to a point, beyond which a further increase cannot be seen, suggesting a working Frank–Starling mechanism in this context similar to that for LA volumes. Similar associations are present for the peak LA-GLS as well.

## 1. Introduction

In recent years, the study of disease-related volumetric and functional abnormalities of the heart chambers has focused on non-invasive imaging methods, primarily modern echocardiographic techniques [1,2,3]. However, in order to understand these abnormalities, more emphasis needs to be placed on understanding the associations between the heart chambers even in healthy circumstances. Three-dimensional speckle-tracking echocardiography (3DSTE) seems to be an optimal method for such physiologic examinations, due to its ability to simultaneous assess the left ventricle (LV) and atrium (LA) at the same time during the same analysis [4,5,6,7,8]. The complex relationship between the LV and LA volumes and functional properties has been shown in recent studies, even by 3DSTE-derived ones [9,10,11,12,13]. A better understanding of the LV volumetric dependence on the systolic LA function represented by peak LA (reservoir) global strains in healthy circumstances could complete this knowledge [14,15]. Therefore, 3DSTE was used for the simultaneous evaluation of these parameters in healthy adults, aiming to examine their complex relationship.

## 2. Methods

### 2.1. Subject Population

The present study comprised 165 healthy individuals with a mean age of 33.1 ± 12.3 years and 90 males. All subjects were non-smokers in sinus rhythm without any known disease or pathological condition; none of them used any drugs, were obese, or athletes. In all cases, physical examination, laboratory tests, standard 12-lead electrocardiography (ECG), and two-dimensional Doppler echocardiography extended with 3DSTE were performed. The present retrospective cohort study is part of the **M**otion **A**nalysis of the heart and **G**reat vessels b**Y** three-dimension**A**l speckle-t**R**acking echocardiography in **Healthy** subjects **(MAGYAR-Healthy) Study,** which has been organized by the University of Szeged for physiologic studies (‘Magyar’ means ‘Hungarian’ in Hungarian language). This study was conducted in accordance with the Declaration of Helsinki (as revised in 2013), approved by the Institutional and Regional Human Biomedical Research Committee of University of Szeged, Hungary (No.: 71/2011 and updated versions), and all individuals gave their informed consent.

### 2.2. Two-Dimensional Doppler Echocardiography

In all cases, a full examination was carried out in accordance with professional guidelines, which included chamber quantifications and determination of routine dimensions, Doppler exclusion of significant regurgitations and stenosis on any valves, and measurement of early (E) and late (A) diastolic mitral inflow velocities and their ratio (E/A) [16]. The same Toshiba Artida^TM^ echocardiography system (Toshiba Medical Systems, Tokyo, Japan) attached to a PST-30BT (1–5 MHz) phased-array transducer was used on all individuals. 

### 2.3. Three-Dimensional Speckle-Tracking Echocardiography--Derived Data Acquisition

The 3DSTE studies were performed in two steps using the same Artida equipment [17,18,19]. After replacing the transducer with a PST-25SX matrix transducer with 3D capability, it was positioned on man/woman’s chest for optimal data acquisition from the apical window. Three-dimensional echocardiographic datasets were acquired during a breath–hold and within six cardiac cycles. Acquired six subvolumes were then automatically merged by the software into one full volume ‘pyramid-shape’ 3D echocardiographic dataset. Data analyses were performed by vendor-provided 3D Wall Motion Tracking software version 2.7 (Ultra Extend, Toshiba Medical Systems, Tokyo, Japan) at a later date.

### 2.4. Three-Dimensional Speckle-Tracking Echocardiography-Derived LV Volumetric Measurements

**Figure 1:**
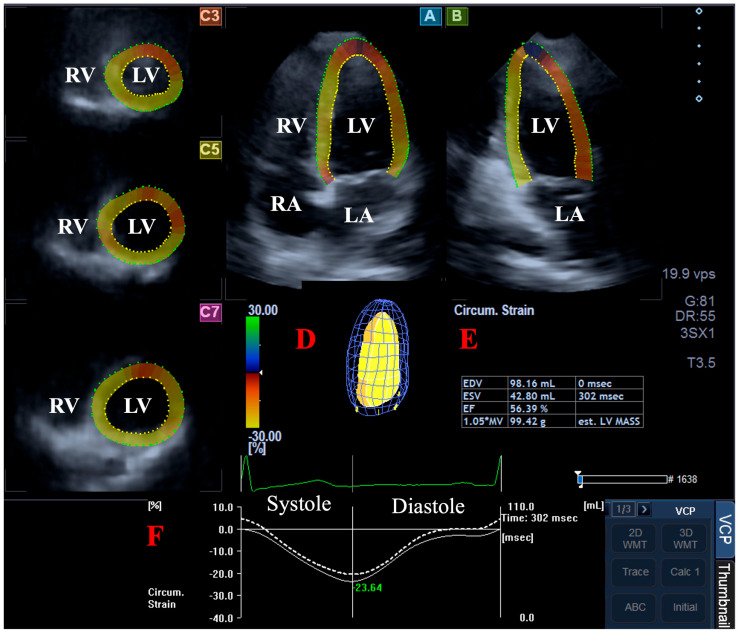
Volumetric assessment of the left ventricle (LV) by three-dimensional (3D) speckle-tracking echocardiography: apical four-chamber view (**A**); apical two-chamber view (**B**); and short-axis view at apical (**C3**), midventricular (**C5**), and basal LV levels (**C7**) are shown together with a virtual 3D model of the LV (**D**) and calculated LV volumes (**E**). Time–global LV circumferential strain curve (white line) and time–LV volume change curve (dashed white line) are shown as well (**F**). Abbreviations: LA, left atrium; LV, left ventricle; RA, right atrium; RV, right ventricle; EDV, end-diastolic volume; ESV, end-systolic volume; EF, ejection fraction.

All datasets were managed by the same software [17]. Following automatic presentation of data in apical apical 2-chamber (AP2CH) and 4-chamber (AP4CH) long-axis views, and apical, midventricular, and basal cross-sectional short-axis views, images were optimised and focused on the LV. After defining the mitral annular (MA)–LV edges and the endocardial LV apical surface, a sequential analysis was started, leading to the build-up of a virtual 3D cast of the LV (Figure 1). Using this model, end-diastolic (EDV) and end-systolic (ESV) LV volumes, LV ejection fraction (EF), and LV mass were calculated.

### 2.5. Three-Dimensional Speckle-Tracking Echocardiography-Derived Assessment of Peak LA Global Strains

**Figure 2:**
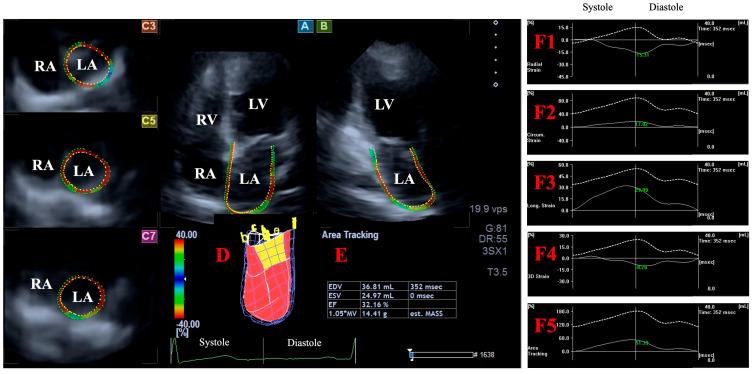
Left atrial (LA) volume and strain assessment by three-dimensional (3D) speckle-tracking echocardiography: apical four-chamber view (**A**); apical two-chamber view (**B**); short-axis view at basal (**C3**), midatrial (**C5**), and superior left atrial levels (**C7**) are shown together with a virtual 3D model of the LA (**D**) and calculated LA volumes (**E**). Time–global LA radial (**F1**), circumferential (**F2**), longitudinal (**F3**), 3D (**F4**), and area (**F5**) strain curves (white lines) and time–LA volume change curve (dashed white lines) are shown as well. LA, left atrium; LV, left ventricle; RA, right atrium; RV, right ventricle; EDV, end-diastolic volume; ESV, end-systolic volume; EF, ejection fraction.

A similar LA analysis was performed on LA-focused images using the same protocol for AP4CH and AP2CH long-axis views and 3 short-axis views in basal, midatrial, and superior regions of the LA [18,19]. Similarly to LV, a 3D LA cast was created, but reference points in AP4CH and AP2CH were determined by defining the endocardial surface of LA between the lateral and septal edges of the MA-LA including the apex of LA, then sequential analysis was performed. Pulmonary veins and LA appendage were excluded from the assessments. Systolic peak LA global (reservoir) strains were defined as the first peak on the twin-peak time-specific global strain curves (Figure 2).

Together with LA volumes, peak LA global strains were also determined using the same virtual LA model:

- LA-Vmax—maximum LA volume at end-systole;
- LA-VpreA—pre-atrial contraction LA volume at early diastole;
- LA-Vmin—minimum LA volume at end-diastole;
- LA-GRS—LA global radial strain representing LA thinning/thickening;
- LA-GCS—LA global circumferential strain representing LA widening/narrowing;
- LA-GLS—LA global longitudinal strain representing LA lengthening/shortening;
- LA-G3DS—LA global 3D strain, combination of all unidirectional LA strains;
- LA-GAS—LA global area strain, combination of LA circumferential and longitudinal strains.

### 2.6. Statistical Analysis

Continuous variables were presented as mean ± standard deviation (SD), while number (%) format was used for the demonstration of categorical data. Independent sample *t*-tests and analysis of variance (ANOVA) tests were performed for group comparisons, where appropriate. For intraobserver and interobserver correlations, intraclass correlation coefficients (ICCs) were calculated. *p* < 0.05 was considered to be statistically significant. SPSS software version 22 (SPSS Inc., Chicago, IL, USA) was used for statistical analyses.

## 3. Results

### 3.1. Two-Dimensional Echocardiography with Doppler

The routine parameters proved to be normal in the cases including the LA diameter measured in the parasternal long-axis view (36.6 ± 4.1 mm), LV end-diastolic diameter (48.2 ± 3.5 mm) and volume (106.9 ± 23.1 mL), LV end-systolic diameter (32.0 ± 3.3 mm) and volume (36.5 ± 9.2 mL), interventricular septum (9.0 ± 1.5 mm) and LV posterior wall (9.1 ± 1.7 mm), and LV-EF (65.7 ± 5.1%). The mean E/A proved to be 1.34 ± 0.40. No healthy individuals had a greater than/equal to grade 1 regurgitation or had significant stenosis on any valves.

### 3.2. Classification of Subjects

The 3DSTE-derived LV volumes and peak LA global strains are shown in Table 1. The LV-EDV, LV-ESV, and peak LA-GRS, LA-GCS, and LA-GLS were measured as the mean ± SD. The subjects were then classified into three subgroups based on these parameters, the cut-off values were mean–SD (63.2 mL, 25.8 mL, 6.7%, 18.3%, and 17.2%, respectively) and mean + SD (108.8 mL, 46.6 mL, 22.9%, 48.9%, and 35.4%, respectively).

### 3.3. Higher LV Volumes vs. Other LV and LA Volumetric Parameters

With an increase in the LV-EDV, a simultaneous increase in the LV-ESV and LV mass could be detected with preserved LV-EF, and a simultaneous increase in the LA-Vmax and LA-VpreA could be detected with preserved LA-Vmin. With an increased LV-ESV, a simultaneous increase in the LV-EDV, LV mass, and all LA volumes could be detected with a reduction in the LV-EF (Table 2).

### 3.4. Higher LV Volumes vs. Peak LA Global Strains

The peak LA-GRS, LA-GLS, and LA-G3DS were increased in the subjects with a mean LV-EDV as compared to those cases with a lower-than-mean LV-EDV. In the cases with a higher-than-mean LV-EDV, no further increase in these peak LA global strains could be detected. The peak LA-GCS and LA-GAS showed tendentious (non-significant) increases with an increasing LV-EDV. The peak LA strains showed similar non-significant associations with the LV-ESV (except for the peak LA-G3DS, which proved to be significant) (Table 2).

### 3.5. Higher Peak LA Global Strains vs. LV and LA Volumes

With an increase in the peak LA-GRS, a non-significant increase in the LV volumes with preserved LV-EF could be detected. The LV mass was the largest in cases of the highest peak LA-GRS. With an increase in the peak LA-GCS, a non-significant increase in the LV-EDV could be detected. The LV-ESV was the smallest in cases of the lowest peak LA-GCS. The LV-EF and LV mass did not change with peak LA-GCS. The LV volumes and LV-EF did not change with peak LA-GLS, while the lowest LV mass was present with the highest peak LA-GLS.

In case of the mean peak LA-GRS, the systolic LA-Vmax was higher when compared to cases with a lower-than-mean peak LA-GRS, and did not show a further increase in cases of a larger-than-mean peak LA-GRS. The diastolic LA volumes (VpreA and Vmin) showed no associations with the peak LA-GRS. The diastolic LA volumes were reduced in the presence of an increased peak LA-GCS and LA-GLS with preserved LA-Vmax (Table 3).

### 3.6. Higher Peak LA Global Strains vs. Other Peak LA Global Strains

With an increase in the peak LA-GRS, the LA-G3DS showed a similar continuously increasing pattern, while the peak LA-GCS, LA-GLS, and LA-GAS were increased in the presence of a mean peak LA-GRS as compared to lower-than-mean values, but a further increase could not be detected in the presence of higher-than-mean values. With an increasing peak LA-GCS, all the peak LA strains showed a similar continuously increasing pattern except for the peak LA-G3DS, which did not increase further when the peak LA-GCS was larger than the mean value. With an increasing peak LA-GLS, the LA-GCS and LA-GAS showed a similar continuously increasing pattern, while the peak LA-G3DS and LA-GRS did not increase further when the peak LA-GLS was larger than the mean value (Table 3).

### 3.7. Reproducibility of 3DSTE-Derived Peak LA Strains and LV Volumes

The mean ± SD differences between the values measured by the two examiners for the LV-EDV, LV-ESV, peak LA-GRS, LA-GLS, LA-GCS, LA-GAS, and LA-G3DS were 1.7 ± 4.4 mL, 1.1 ± 4.4 mL, −1.9 ± 9.9%, 4.1 ± 12.4%, 0.6 ± 8.1%, 10.1 ± 38.1%, and −1.5 ± 10.4%, respectively, with a correlation coefficient between these independent measurements of 0.90 (*p* < 0.01), 0.90 (*p* < 0.01), 0.67 (*p* < 0.01), 0.81 (*p* < 0.01), 0.67 (*p* < 0.01), 0.63 (*p* < 0.01), and 0.67 (*p* < 0.01), respectively (interobserver variability). The mean ± SD differences between the values obtained by the two measurements by examiner 1 for the same parameters were 1.7 ± 6.5 mL, 0.9 ± 5.1 mL, −1.2 ± 10.2%, 3.9 ± 13.9%, 1.4 ± 13.2%, 4.8 ± 34.2%, and 1.1 ± 10.2%, respectively, with a correlation coefficient between these independent measurements of 0.91 (*p* < 0.01), 0.91 (*p* < 0.01), 0.71 (*p* < 0.01), 0.76 (*p* < 0.01), 0.59 (*p* < 0.01), 0.73 (*p* < 0.01), and 0.72 (*p* < 0.01), respectively (intraobserver variability).

## 4. Discussion

Although the LA and LV are integral units, relatively few clinical imaging studies have analysed their volumetric and functional changes simultaneously during a heart cycle in healthy adults [9,10]. The LV fills from the LA, having the largest volume in the end-diastole and the smallest in the end-systole. The LA has a triple function during the cardiac cycle, serving as a reservoir in systole (having the largest volume); a conduit in early diastole, allowing for blood to flow from the veins to the LV; and a booster pump in late diastole (having the smallest volume) [11,12,13,20]. One might wonder what relationship could be confirmed between the LV volumes and LA strains, which are quantitative features of the LA wall contractility measured in systole in healthy adults [21,22].

In recent decades, non-invasive imaging has undergone tremendous technical development. Not only have cardiac magnetic resonance imaging and computer tomography become routine procedures, but new echocardiographic methods, including 3DSTE, have become part of the imaging options [4,5,6,7,8]. Three-dimensional speckle-tracking echocardiography can even be considered as the most modern echocardiographic method since, with the help of a virtual 3D model, it is possible to perform volumetric and strain measurements of a given heart cavity at the same time. Both 3DSTE-derived LV volumetric [23,24,25,26] and LA volume/strain assessments have been validated [25,27,28], and normal references are also available [17,18,19].

This is the first study to analyse the associations between 3DSTE-derived LV volumes and systolic peak LA (reservoir) global strains at the same time in healthy adults. In a recent study from the MAGYAR-Healthy Study, increased LV volumes were found to be associated with increased LA volumes in healthy adults [9], while peak LA-GRS and LA-G3DS were found to be increased with larger LA volumes only up to a point, beyond which this association disappeared, suggesting a working Frank–Starling mechanism [10]. The present analysis goes further by examining the associations between systolic peak LA global strains and LV volumes. It was detected that not only the peak LA-GRS and peak LA-G3DS increased with a larger LV-EDV up to a point, but the peak LA-GLS did as well. Moreover, the peak LA-GCS and LA-GAS showed tendentious (non-significant) increases with the LV-EDV. Only the peak LA-G3DS showed a similar association with the LV-ESV. Moreover, an increase in the peak LA strains showed a tendentious increase in the LV volumes with a preserved LV-EF. These results suggest a working Frank–Starling mechanism, but the associations are more complex than that for only the LA, and other factors need to be considered in the context of the relationship between the LV and the LA.

Although the LA wall is thinner and its myocardial architecture is more simple than that of the LV, the complexity of the LA contraction–relaxation pattern in opposition to that of the LV is suggested by the 3DSTE-derived strain analysis. The muscle fibres of the walls of the LA consist of one or more alternating layers with marked cross-sectional variations in thickness, described as circumferential (parallel to the mitral annulus) or longitudinal (running perpendicular to the plane of the mitral orifice). Based on these facts, there could be a debate about whether it makes sense to talk about the LA-RS (and consequently the LA-3DS) in addition to the LA-LS and LA-CS (and consequently the LA-AS); as the wall of the LA is thin, this fact may induce further investigations to clarify this [28]. The present study points our attention as well to the fact that more emphasis should be placed on investigating the physiologic relationship between the volumes and functional properties of certain heart chambers, even in non-healthy circumstances, for examining the pathophysiologic background of developing heart failure and related volumetric and functional abnormalities.

### Limitations Section

The most important ones are listed here:

− The image quality which can be attained during 2D echocardiography is still higher than that of 3DSTE due to its higher spatial and temporal resolution [4,5,6,7,8].
− For optimal image quality, six wedge-shaped subvolumes during six cardiac cycles were acquired. However, this technical requirement required great care, which may have affected the image quality.
− Under current technical conditions, subjects have to be in sinus rhythm in order to be able to be examined by 3DSTE [4,5,6,7,8]. However, this was not an issue in this study, due to the fact that healthy subjects were examined.
− All the subjects were considered to be healthy, but it could not be excluded with 100 percent certainty that the examined adults did not have an undiagnosed latent disease. Further examinations could have strengthened our findings.
− The present study did not aim to validate the above-mentioned parameters due to their previously validated nature [23,24,25,26,27,28].
− Other parameters of the LA/LV, and the volumes and strains of the other heart chambers, were not purposed to be analysed [29,30,31,32].

## 5. Conclusions

In healthy adults, the 3DSTE-derived peak LA-GRS and LA-G3DS increased with a larger LV-EDV up to a point, beyond which a further increase could not be detected, suggesting a working Frank–Starling mechanism in this context, similarly to the LA volumes. Similar associations were present for the peak LA-GLS as well.

## Figures and Tables

**Table 1:** Three-dimensional speckle-tracking echocardiography-derived left ventricular volumes and peak left atrial (reservoir) global strains.

Parameters	Measures
Left ventricular volumes	
end-diastolic left ventricular volume (LV-EDV)	86.0 ± 22.8
end-systolic left ventricular volume (LV-ESV)	36.2 ± 10.4
left ventricular ejection fraction (LV-EF)	58.2 ± 5.6
left ventricular mass (g)	158.9 ± 32.0
Peak left atrial (reservoir) global strains	
peak left atrial global radial strain (LA-GRS, %)	−14.8 ± 8.1
peak left atrial global circumferential strain (LA-GCS, %)	33.6 ± 15.3
peak left atrial global longitudinal strain (LA-GLS, %)	26.3 ± 9.1
peak left atrial global 3D strain (LA-G3DS, %)	−7.5 ± 5.9
peak left atrial global area strain (LA-GAS, %)	67.5 ± 28.1

**Table 2:** Left ventricular volumes and peak left atrial global strains in different left ventricular volume groups.

	LV-EDV ≤ 63.2 mL (n = 19)	63.2 mL < LV-EDV < 108.8 mL (n = 128)	108.8 mL ≤ LV-EDV (n = 18)	LV-ESV ≤ 25.8 mL (n = 22)	25.8 mL < LV-ESV < 46.6 mL (n = 121)	46.6 mL ≤ LV-ESV (n = 22)
LV-EDV (mL)	53.6 ± 12.3	84.7 ± 11.9 *	131.2 ± 19.6 *†	64.1 ± 14.2	83.6 ± 12.8 #	125.0 ± 22.1 #‡
LV-ESV (mL)	23.9 ± 5.7	35.4 ± 6.6 *	56.1 ± 8.9 *†	21.8 ± 3.3	35.4 ± 5.5 #	54.9 ± 8.1 #‡
LV-EF (%)	58.7 ± 8.0	58.2 ± 5.4	57.1 ± 4.3	65.4 ± 6.1	57.4 ± 5.0 #	55.7 ± 3.9 #
LV mass (g)	133.3 ± 23.7	157.6 ± 28.8 *	196.2 ± 29.5 *†	134.5 ± 25.2	157.2 ± 28.3 #	191.9 ± 29.4 #‡
LA-Vmax (mL)	33.2 ± 9.8	40.7 ± 12.7 *	49.0 ± 14.0 *†	34.1 ± 12.0	40.7 ± 12.1 #	50.0 ± 15.0 #‡
LA-VpreA (mL)	24.3 ± 9.0	27.3 ± 11.6	33.3 ± 13.9 *†	23.8 ± 9.5	27.4 ± 11.3	34.5 ± 14.2 #‡
LA-Vmin (mL)	18.1 ± 6.4	19.2 ± 8.3	21.9 ± 8.2	16.5 ± 5.2	19.1 ± 7.8	23.7 ± 10.0 #‡
LA-GRS (%)	−10.0 ± 5.7	−15.5 ± 8.3 *	−15.3 ± 7.4 *	−12.2 ± 9.3	−15.5 ± 8.1	−14.7 ± 7.3
LA-GCS (%)	29.1 ± 16.0	33.3 ± 14.4	41.2 ± 18.2	32.5 ± 22.2	33.6 ± 13.7	36.2 ± 18.6
LA-GLS (%)	21.2 ± 6.0	26.9 ± 9.3 *	30.1 ± 7.1 *	25.4 ± 10.1	26.5 ± 8.7	26.3 ± 10.6
LA-G3DS (%)	−3.4 ± 2.7	−7.9 ± 6.0 *	−8.3 ± 6.0 *	−4.6 ± 4.3	−7.9 ± 6.1#	−8.4 ± 5.9 #
LA-GAS (%)	58.2 ± 29.4	67.0 ± 26.7	79.1 ± 34.8 *	65.9 ± 38.3	67.1 ± 25.3	74.3 ± 35.9

* *p* < 0.05 vs. LV-EDV ≤ 63.2 mL; † *p* < 0.05 vs. 63.2 mL < LV-EDV < 108.8 mL; # *p* < 0.05 vs. LV-ESV ≤ 25.8 mL; ‡ *p* < 0.05 vs. 25.8 mL < LV-ESV < 46.6 mL. **Abbreviations:** LV = left ventricular; ESV = end-systolic volume; EDV = end-diastolic volume; EF = ejection fraction; VpreA = early diastolic pre-atrial contraction LA volume; Vmax = end-systolic maximum LA volume; Vmin = end-diastolic minimum LA volume; LA = left atrial; G3DS = global three-dimensional strain; GRS = global radial strain; GCS = global circumferential strain; GLS = global longitudinal strain; GAS = global area strain.

**Table 3:** Left ventricular volumes and peak left atrial global strains in different peak left atrial global strain groups.

	LA-GRS ≤ −6.7% (n = 24)	−6.7% < LA-GRS < −22.9% (n = 121)	−22.9% ≤ LA-GRS (n = 20)	LA-GCS ≤ 18.3% (n = 25)	18.3% < LA-GCS < 48.9% (n = 112)	48.9% ≤ LA-GCS (n = 28)	LA-GLS ≤ 17.2% (n = 25)	17.2% < LA-GLS < 35.4% (n = 115)	35.4% ≤ LA-GLS (n = 25)
LV-EDV (mL)	82.3 ± 25.3	86.1 ± 21.2	93.3 ± 22.1	79.5 ± 24.8	86.7 ± 21.0	91.5 ± 22.5	86.3 ± 24.5	86.1 ± 22.6	88.2 ± 16.4
LV-ESV (mL)	34.4 ± 12.3	36.0 ± 9.5	39.6 ± 12.6	32.1 ± 11.3	37.0 ± 9.5 ‡	36.7 ± 12.2	37.6 ± 11.5	35.8 ± 10.6	37.0 ± 8.2
LV-EF (%)	58.9 ± 5−7	58.1 ± 5.2	57.8 ± 7.9	60.2 ± 5.6	57.2 ± 5.0	60.1 ± 7.0	57.1 ± 5.9	58.4 ± 5.8	58.1 ± 4.8
LV mass (g)	154.9 ± 31.3	157.0 ± 31.5	175.1 ± 32.9 *†	156.8 ± 25.2	159.5 ± 32.4	158.2 ± 36.5	166.0 ± 29.9	159.6 ± 31.5	148.5 ± 35.1 $
LA-Vmax (mL)	35.5 ± 13.2	41.5 ± 12.9 *	42.8 ± 13.2	38.4 ± 11.7	41.6 ± 13.0	39.8 ± 14.5	39.8 ± 15.6	41.1 ± 13.1	40.0 ± 10.0
LA-VpreA (mL)	26.1 ± 11.3	27.6 ± 12.1	29.3 ± 10.5	30.1 ± 10.4	28.0 ± 11.6	23.8 ± 13.2 ‡	31.2 ± 14.3	27.7 ± 11.5	23.4 ± 8.8 $
LA-Vmin (mL)	20.2 ± 8.7	19.1 ± 8.3	19.0 ± 6.5	24.5 ± 8.3	19.6 ± 7.9 ‡	13.7 ± 5.2 ‡#	24.0 ± 11.6	19.2 ± 7.2 $	14.9 ± 5.1 $&
LA-GRS (%)	−3.0 ± 2.3	−14.8 ± 4.7 *	−28.9 ± 6.6 *†	−10.0 ± 7.0	−15.1 ± 8.0 ‡	−18.2 ± 7.7 ‡#	−10.2 ± 8.8	−15.6 ± 8.1 $	−15.7 ± 5.6 $
LA-GCS (%)	24.2 ± 10.6	34.7 ± 15.3 *	37.6 ± 16.4 *	13.6 ± 3.6	31.7 ± 7.8 ‡	59.0 ± 10.6 ‡#	21.9 ± 11.9	33.9 ± 14.3 $	43.6 ± 15.5 $&
LA-GLS (%)	19.3 ± 8.6	27.6 ± 8.8 *	26.2 ± 8.0 *	17.8 ± 5.5	26.7 ± 8.2 ‡	31.9 ± 9.7 ‡#	13.2 ± 3.3	25.9 ± 4.8 $	41.0 ± 6.1 $&
LA-G3DS (%)	−0.9 ± 1.1	−7.5 ± 4.3 *	−14.9 ± 8.2 *†	−4.4 ± 4.1	−8.1 ± 6.4 ‡	−7.7 ± 4.2 ‡	−4.3 ± 5.5	−7.8 ± 6.0 $	−9.0 ± 4.4 $
LA-GAS (%)	48.0 ± 25.1	70.9 ± 26.9 *	69.8 ± 30.5 *	31.7 ± 8.4	65.1 ± 17.9 ‡	109.1 ± 20.4 ‡#	35.2 ± 16.6	67.3 ± 22.1 $	100.5 ± 24.0 $&

* *p* < 0.05 vs. LA-GRS ≤ −6.7%, † *p* < 0.05 vs. −6.7% < LA-GRS < −22.9%, ‡ *p* < 0.05 vs. LA-GCS ≤ 18.3%, # *p* < 0.05 vs. 18.3% < LA-GCS < 48.9%, $ *p* < 0.05 vs. LA-GLS ≤ 17.2%, & *p* < 0.05 vs. 17.2% < LA-GLS < 35.4%. **Abbreviations:** LV = left ventricular; ESV = end-systolic volume; EDV = end-diastolic volume; EF = ejection fraction; VpreA = early diastolic pre-atrial contraction LA volume; Vmax = end-systolic maximum LA volume; Vmin = end-diastolic minimum LA volume; LA = left atrial; G3DS = global three-dimensional strain; GCS = global circumferential strain; GRS = global radial strain; GLS = global longitudinal strain; GAS = global area strain.

## Data Availability

All the data are available.
